# Acute Myocardial Infarction and Stage E Shock: Insights From the RECOVER III Study

**DOI:** 10.1016/j.jscai.2024.102462

**Published:** 2025-01-07

**Authors:** Ivan D. Hanson, Andres Palomo, Adam Tawney, Simon R. Dixon, Dana Bentley, Srihari S. Naidu, Mir B. Basir, William W. O’Neill

**Affiliations:** a Department of Cardiovascular Medicine, Corewell Health William Beaumont University Hospital, Royal Oak, Michigan; b Abiomed, Danvers, Massachusetts; c Department of Cardiology, Westchester Medical Center and New York Medical College, Valhalla, New York; d Division of Cardiology, Henry Ford Hospital, Detroit, Michigan

**Keywords:** acute myocardial infarction with cardiogenic shock, mechanical circulatory support, percutaneous coronary intervention, SCAI shock class

## Abstract

**Background:**

The present analysis reports characteristics and outcomes of Society of Cardiovascular Angiography & Interventions (SCAI) stage E shock patients with acute myocardial infarction with cardiogenic shock (AMICS) undergoing percutaneous coronary intervention (PCI) who improved to stage C or D within 24 hours of Impella support (“responders”) vs those patients who remained in stage E (“nonresponders”).

**Methods:**

The SCAI shock stage was assigned prior to initiation of Impella, and a second SCAI shock classification was performed within 24 hours of Impella support. SCAI shock stage was assigned independently by 2 reviewers; in cases where there was a discrepancy, a third reviewer adjudicated the stage assignment. Criteria such as a low pH (≤7.1), the need for multiple vasopressors/mechanical circulatory support devices, or the need for cardiopulmonary resuscitation were used to define stage E shock.

**Results:**

Of the 415 RECOVER III patients, 298 presented in stage E shock; 152 (51.1%) were responders and 145 (48.8%) were nonresponders. Kaplan-Meier 30-day survival estimates were 56.9% and 28.6% in responders and nonresponders, respectively (*P* < .001). In multivariate analysis, fewer inotropic medications during Impella support (*P* < .0001), more lesions treated (*P* = .01), Impella support initiated pre-PCI (*P* = .03), and baseline white blood cell (*P* = .048) were all significant predictors for responsiveness to therapy.

**Conclusions:**

Stage E patients who improved to stage C/D within 24 hours of Impella support had significantly better survival than those who remained in stage E. Predictors of responsiveness to therapy were mostly related to shock treatment strategy, and not baseline characteristics. This suggests that whether stage E patients will improve with Impella support is difficult to determine at the time support is initiated, and the SCAI shock stage should be repeated within 24 hours to more accurately determine the prognosis.

## Introduction

Acute myocardial infarction with cardiogenic shock (AMICS) is associated with poor survival.^1^ Immediate revascularization via percutaneous coronary intervention (PCI) has been shown to salvage myocardium and improve outcomes in this lethal condition, reducing mortality with an absolute risk reduction of 12.8% at 6 months.^2^ The DanGer Shock trial, which randomized patients to Impella vs standard of care, similarly demonstrated improvements in mortality with an absolute risk reduction of 12.7% at 6 months.^3^ DanGer Shock established the importance of Impella as an equally important therapy to revascularization in patients with AMICS, with investigators reporting that the number needed to treat to avoid 1 death was 8.^3^

The Impella devices are transcatheter, transvalvular, and left ventricular axial flow pumps (Abiomed) that are placed in the cardiac catheterization laboratory and can generate up to 4 L/min of microaxial, in-parallel flow, depending on loading conditions. The prospective, nonrandomized National Cardiogenic Shock Initiative (NCSI) study provided a framework for how Impella could be utilized in patients with AMICS. The NCSI demonstrated that a protocolized approach for the delivery and management of Impella was associated with a 71% survival to discharge.^4^ This study also validated the value of the Society of Cardiovascular Angiography & Interventions (SCAI) shock classification system in AMICS patients treated with Impella.^5^ Both the DanGer Shock trial and the NCSI required patients included in these respective studies to fit into a set of inclusion and exclusion criteria. Most notably, both studies excluded patients with unwitnessed or prolonged cardiac arrest.

Since Food and Drug Administration approval of the Impella CP device in April 2016, an Food and Drug Administration-mandated postapproval study (RECOVER III) has captured real-world, all-comer outcomes of AMICS patients treated with Impella who received revascularization.^6^ This single-arm, multicenter, prospective, observational study enrolled primarily stage E shock patients (72.2%) pre-Impella and demonstrated an overall 30-day mortality rate of 55.6%. Although pre-Impella shock severity was associated with mortality, the shock stage within 24 hours after Impella initiation demonstrated a more significant association, with stage E shock at 24 hours being the only shock stage significantly predictive of 30-day mortality.^6^

We now report characteristics and outcomes of RECOVER III stage E shock patients who improved to stage C or D within 24 hours of Impella support (“responders”) vs those patients who remained in stage E (“nonresponders”).

## Methods

### Study design and population

Details of the RECOVER III study have been published previously.^6^ Briefly, RECOVER III is an observational, prospective, postapproval study nested within the cVAD registry (ClinicalTrials.gov, NCT04136392). Institutional review boards/independent ethics committees approved the study protocol at all study sites, and the study was conducted in accordance with the Declaration of Helsinki. Patients enrolled in this study underwent revascularization with Impella support initiated prerevascularization, during revascularization, or postrevascularization, with the vast majority of the 418 enrolled patients undergoing PCI. All patients provided informed consent for prospective follow-up data collection, though a study amendment allowed waivers of informed consent where patients did not actively decline consent.

We previously performed SCAI shock classification for the RECOVER III study population, both at baseline (pre-Impella) and at a second timepoint within 24 hours of Impella placement.^6^ The majority of patients (72%) were classified as those in shock stage E at pre-Impella assessment, using a SCAI shock classification scheme similar to the one we employed when performing SCAI shock classification on the NCSI population,^5^ with a few adjustments to allow for the real-world nature of the cVAD registry, where invasive hemodynamic data such as cardiac indices were not frequently obtained, and granular data regarding pre-Impella cardiac arrest were often lacking. Full details of the SCAI classification and methodology used were detailed previously.^6^ Briefly, a medical record review was conducted in order to provide supplemental data to perform SCAI shock stage classification, beyond what was recorded in the cVAD database. The Cardiogenic Shock Working Group-SCAI criteria for SCAI shock staging were followed whenever possible.^7^ The same classification scheme (either SCAI shock stage or Cardiogenic Shock Working Group-SCAI shock stage) was used for baseline assessment and reassessment for each patient. Owing to the data limitations of the cVAD registry regarding duration of cardiac arrest and postarrest status, both in-hospital cardiac arrest and out-of-hospital cardiac arrest were considered as criteria for stage E shock, though patients with in-hospital cardiac arrest demonstrated at least 1 other factor for stage E to be classified as such. Case report forms are displayed in Supplemental Figures S1 and S2.

In this post hoc, subgroup analysis, we aimed to assess the 298 patients classified as those in stage E shock pre-Impella. Approximately half of the patients who were classified as those in stage E shock pre-Impella improved to stage C/D at 24 hours, whereas half did not improve. We sought to compare baseline, admission, and procedural characteristics, as well as clinical outcomes, of patients who “responded” to therapy (ie, the responders cohort) and patients who did not respond (the nonresponders cohort). We further sought to investigate whether any baseline, admission, or procedural characteristics were predictive of responsiveness to therapy.

### End points and statistical analysis

Major adverse cerebrovascular and cardiac events were reported through hospital discharge, 30 days, 90 days, and 1 year, and comprised mortality, myocardial infarction, cerebrovascular accident/stroke/transient ischemic attack, and coronary revascularization/emergent coronary artery bypass grafting. Kaplan-Meier survival curve analysis generated survival estimates through 30 days for the responder and nonresponder cohorts.

Univariate and multivariable regression analyses were performed to identify predictors for improvement in the SCAI shock stage (at 24 hours). A forward fit approach was used, with all variables associated with a *P* value <.15 in univariate analyses included in the multivariable model, with the exception of variables with significant data missingness. Statistical analyses were performed with SAS version 9.4 (SAS Institute). All *P* values were 2-tailed; *P* < .05 was considered statistically significant.

## Results

### Baseline and procedural characteristics

Of 298 patients classified as those in stage E shock at pre-Impella assessment, 152 patients (51%) improved in the SCAI shock stage within 24 hours of Impella placement (the responder cohort), and 145 patients (49%) remained in stage E (the nonresponder cohort; Central Illustration). There were no significant differences in baseline demographics or medical history between responders and nonresponders (Table 1). Responders had lower white blood cell (WBC) count, higher pH, and lower heart rate than nonresponders (Table 2). Lactate was also numerically lower in responders.

**Central Illustration:**
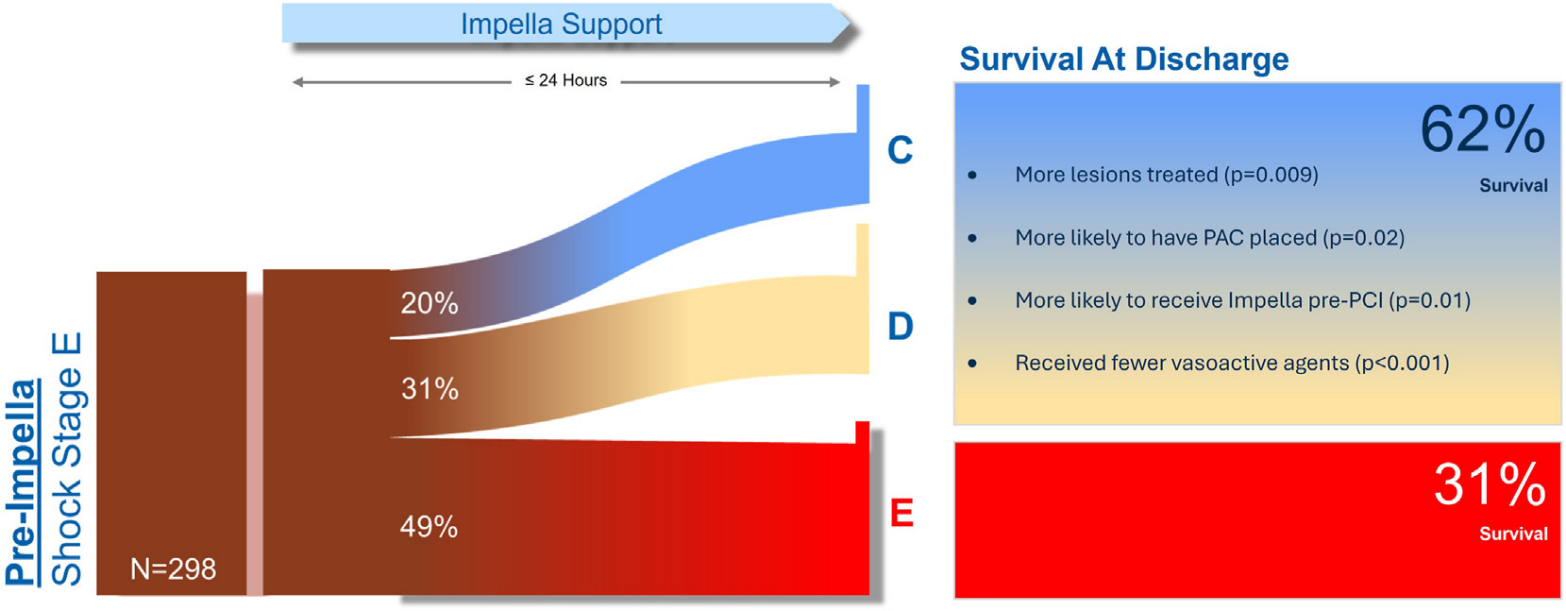
**Visualization of change in the Society of Cardiovascular Angiography & Interventions (SCAI) shock stage from baseline to ≤24-hour assessment, in patients classified as those in SCAI shock stage E at baseline assessment.** A total of 51% of SCAI shock stage E patients improved in the SCAI shock stage within 24 hours of initiation of Impella support (ie, “responders”) and 49% remained in stage E (“nonresponders”). Survival at discharge in responders and nonresponders was 62% and 31%, respectively. PCI, percutaneous coronary intervention.

**Table 1:** Baseline demographics and medical history in SCAI stage E patients, responders, and nonresponders.

	SCAI stage E (N = 298)	Responder (n = 152)	Nonresponder (n = 145)	*P* value
Age, y	63.7 ± 11.37 (298)	64.0 ± 11.44 (152)	63.5 ± 11.33 (145)	.72
Male sex	76.2% (227/298)	79.6% (121/152)	72.4% (105/145)	.15
Race				
White	70.1% (209/298)	66.4% (101/152)	73.8% (107/145)	.17
Black or African American	13.4% (40/298)	16.4% (25/152)	10.3% (15/145)	.12
Asian	3.7% (11/298)	3.3% (5/152)	4.1% (6/145)	.70
American Indian or Alaska Native	0.3% (1/298)	0% (0/152)	0.7% (1/145)	.31
Other	2.3% (7/298)	3.3% (5/152)	1.4% (2/145)	.28
Unknown	10.1% (30/298)	10.5% (16/152)	9.7% (14/145)	.80
Body surface area, m^2^	2.0 ± 0.24 (287)	2.0 ± 0.23 (150)	2.0 ± 0.24 (136)	.42
LVEF, %	26.4 ± 12.99 (141)	25.7 ± 12.37 (70)	27.0 ± 13.62 (71)	.54
History of CAD	45.6% (125/274)	47.2% (68/144)	44.2% (57/129)	.62
Diabetes	45.2% (123/272)	44.3% (62/140)	46.6% (61/131)	.71
Smoking	57.3% (149/260)	55.8% (77/138)	59.5% (72/121)	.55
Hypertension	77.3% (215/278)	80.4% (115/143)	73.9% (99/134)	.19
Prior MI	24.3% (64/263)	25.5% (35/137)	23.2% (29/125)	.66
Prior PCI	27.3% (76/278)	28.1% (41/146)	26.7% (35/131)	.80
Prior CABG	7.8% (22/282)	9.4% (14/149)	6.1% (8/132)	.30
Arrhythmia	15.0% (37/247)	18.5% (25/135)	10.8% (12/111)	.09
Prior stroke/TIA	8.1% (22/273)	10.5% (15/143)	5.4% (7/129)	.13
Chronic pulmonary disease	13.9% (37/266)	11.5% (16/139)	16.7% (21/126)	.23
Renal insufficiency	16.4% (44/269)	18.3% (26/142)	14.3% (18/126)	.37
Prior ICD implanted	3.1% (8/260)	3.6% (5/140)	2.5% (3/119)	.63

Patient and procedural characteristics are presented for the overall cohort of 298 patients classified as those in SCAI stage E at baseline assessment, as well as within the “responder” and “nonresponder” subgroups, with 1 patient lacking data for a second SCAI classification and not included in either subgroup.

Categorical data are expressed as percentage (numerator/denominator). Continuous data are expressed as mean ± SD (denominator).

CABG, coronary artery bypass grafting; CAD, coronary artery disease; ICD, implantable cardioverter defibrillator; LVEF, left ventricular ejection fraction; MI, myocardial infarction; PCI, percutaneous coronary intervention; TIA, transient ischemic attack.

**Table 2:** Baseline laboratory values and hemodynamics.

	SCAI stage E (N = 298)	Responder (n = 152)	Nonresponder (n = 145)	*P* value
White blood cells, K/mm^3^	14.8 ± 8.54 (235)	13.3 ± 5.98 (123)	16.4 ± 10.50 (111)	.006
Platelets, K/mm^3^	243.2 ± 105.9 (232)	250.7 ± 115.9 (122)	236.0 ± 93.11 (109)	.29
Hemoglobin, g/dL	12.7 ± 2.59 (242)	12.6 ± 2.72 (126)	12.8 ± 2.46 (115)	.71
Hematocrit, %	38.3 ± 7.45 (219)	37.7 ± 7.54 (118)	39.0 ± 7.31 (100)	.21
BUN, mg/dL	24.2 ± 14.73 (221)	23.9 ± 14.14 (116)	24.7 ± 15.43 (104)	.70
Serum creatinine, mg/dL	2.0 ± 4.44 (233)	1.6 ± 1.05 (121)	2.4 ± 6.32 (111)	.16
Total bilirubin, mg/dL	0.7 ± 0.59 (135)	0.7 ± 0.37 (70)	0.8 ± 0.76 (65)	.40
pH^a^	7.1 ± 0.56 (176)	7.2 ± 0.14 (88)	7.0 ± 0.78 (87)	.03
Lactate, mmol/L^a^	9.2 ± 7.59 (145)	8.0 ± 4.98 (69)	10.4 ± 9.29 (75)	.05
Heart rate, bpm	96.8 ± 28.04 (272)	93.5 ± 25.57 (137)	100.2 ± 30.17 (134)	.048
SBP, mm Hg	109.4 ± 30.07 (266)	109.3 ± 28.84 (138)	109.5 ± 31.56 (127)	.96
DBP, mm Hg	68.3 ± 22.60 (266)	67.9 ± 21.79 (138)	68.6 ± 23.62 (127)	.81
MAP, mm Hg	82.3 ± 24.87 (270)	81.9 ± 23.25 (139)	82.7 ± 26.66 (130)	.80

Values are mean ± SD (denominator).

BUN, blood urea nitrogen; DBP, diastolic blood pressure; MAP, mean arterial pressure; SBP, systolic blood pressure.

a Values derived from chart review.

Admission and procedural characteristics are presented in Tables 3 and 4, respectively. More responders than nonresponders had a shock for >24 hours prior to Impella (7.7% vs 2.1%; *P* = .0314). Responders were more likely to have pulmonary artery catheterization (90.8% vs 81.1%; *P* = .016). Responders were on fewer vasoactive agents compared to nonresponders (2.0 vs 2.5; *P* < .001), more often had Impella implanted prior to PCI (63.5% vs 48.6%; *P* = .0106), and had higher maximum flow rate (“P-level” 8.0 vs 6.7; *P* = .0085). Responders were less likely to have additional mechanical circulatory support devices implanted within 24 hours of initial Impella placement (6.6% vs 26.2%; *P* < .0001) or to require vasoactive agents in the first 24 hours of Impella support (57.9% vs 86.2%; *P* < 0.001). Responders also had a significantly longer hospital stay (14.2 days vs 10.1 days; *P* = .005).

**Table 3:** Admission characteristics.

	SCAI stage E (N = 298)	Responder (n = 152)	Nonresponder (n = 145)	*P* value
Transferred from another hospital	41.2% (100/243)	38.8% (47/121)	43.8% (53/121)	.43
Cardiogenic shock on admission	69.0% (169/245)	69.2% (83/120)	68.5% (85/124)	.92
Approximate duration of shock from onset to Impella support^a^				
<6 h	75.0% (213/284)	69.9% (100/143)	80.0% (112/140)	.05
6-12 h	5.3% (15/284)	5.6% (8/143)	5.0% (7/140)	.82
12-24 h	3.9% (11/284)	3.5% (5/143)	4.3% (6/140)	.73
>24 h	4.9% (14/284)	7.7% (11/143)	2.1% (3/140)	.03
Unknown	11.5% (31/269)	13.8% (19/138)	9.2% (12/130)	.25
Approximate duration of shock from onset to Impella support (nontransfer patients only)^a^				
<6 h	78.3% (108/138)	70.4% (50/71)	86.4% (57/66)	.02
6-12 h	5.1% (7/138)	5.6% (4/71)	4.5% (3/66)	.77
12-24 h	2.2% (3/138)	1.4% (1/71)	3.0% (2/66)	.52
>24 h	2.9% (4/138)	5.6% (4/71)	0% (0/66)	.05
Unknown	11.6% (16/138)	16.9% (12/71)	6.1% (4/66)	.048
Hypoxic-ischemic brain injury prior to Impella (first Impella)	9.5% (20/211)	7.4% (9/121)	12.4% (11/89)	.23
Cardiac arrest^b^				
Out-of-hospital cardiac arrest	36.1% (107/296)	34.7% (52/150)	37.2% (54/145)	.65
In-hospital cardiac arrest prior to Impella	57.0% (170/298)	55.3% (84/152)	59.3% (86/145)	.48
Any CPR^b^	82.2% (245/298)	78.9% (120/152)	85.5% (124/145)	.14
AMI	100.0% (298/298)	100.0% (152/152)	100.0% (145/145)	–
STEMI	80.6% (225/279)	78.1% (107/137)	83.0% (117/141)	.30
NSTEMI	19.4% (54/279)	21.9% (30/137)	17.0% (24/141)	.30

Categorical data are expressed as percentage (numerator/denominator). Continuous data are expressed as mean ± SD (denominator).

AMI, acute myocardial infarction; CPR, cardiopulmonary resuscitation; NSTEMI, non–ST-elevation myocardial infarction; STEMI, ST-elevation myocardial infarction.

a Approximate duration of shock from onset to Impella support was captured in the cVAD database with the above-listed categories.

b Values derived from chart review.

**Table 4:** Treatment characteristics.

	SCAI stage E (N = 298)	Responder (n = 152)	Nonresponder (n = 145)	*P* value
Patients required inotropes/vasopressors prior to Impella^a^	84.8% (251/296)	83.3% (125/150)	86.2% (125/145)	.49
If yes, maximum No. of different inotropes/vasopressors^a^	2.2 ± 1.03 (251)	2.0 ± 0.95 (125)	2.5 ± 1.03 (125)	<.0001
1	27.5% (69/251)	36.8% (46/125)	17.6% (22/125)	–
2	33.9% (85/251)	36.8% (46/125)	31.2% (39/125)	–
3+	38.6% (97/251)	26.4% (33/125)	51.2% (64/125)	–
MCS (IABP, VAD, ECMO) used prior to Impella^a^	22.2% (66/297)	21.9% (33/151)	22.8% (33/145)	.85
Patient supported with IABP prior to Impella^a^	18.2% (54/297)	18.5% (28/151)	17.9% (26/145)	.89
Swan-Ganz/PA catheter	86.1% (255/296)	90.8% (138/152)	81.1% (116/143)	.02
Door-to-support time <90 min	30.6% (78/255)	31.3% (42/134)	30.0% (36/120)	.82
Impella pre-PCI	56.4% (163/289)	63.5% (94/148)	48.6% (68/140)	.01
Impella CP use	91.3% (272/298)	89.5% (136/152)	93.1% (135/145)	.27
Highest Impella P level	7.6 ± 1.56 (80)	8.0 ± 0.58 (54)	6.7 ± 2.38 (26)	.009
Highest pump flow, L/min	3.6 ± 1.53 (230)	3.6 ± 1.46 (120)	3.7 ± 1.62 (109)	.57
Duration of Impella support, h	66.9 ± 68.23 (224)	70.6 ± 58.93 (117)	63.4 ± 77.35 (106)	.44
Duration of index PCI procedure, h	1.7 ± 1.10 (245)	1.7 ± 0.95 (125)	1.7 ± 1.24 (119)	.83
Contrast volume, mL	207.5 ± 109.4 (252)	208.7 ± 110.3 (132)	206.6 ± 109.2 (119)	.87
Patients required inotropes/vasopressors during Impella support (24 h)^a^^,^^b^	71.7% (213/297)	57.9% (88/152)	86.2% (125/145)	<.001
If yes, maximum No. of different inotropes/vasopressors^a^	2.3 ± 1.17 (213)	1.4 ± 0.49 (88)	3.0 ± 1.08 (125)	<.001
1	30.5% (65/213)	59.1% (52/88)	10.4% (13/125)	–
2	27.2% (58/213)	40.9% (36/88)	17.6% (22/125)	–
3+	42.3% (90/213)	0.0% (0/88)	72.0% (90/125)	–
Additional devices implanted post-Impella placement (24 h)^a^^,^^b^	16.2% (48/297)	6.6% (10/152)	26.2% (38/145)	<.001
Second Impella implanted	8.7% (26/298)	7.9% (12/152)	9.7% (14/145)	.59
Impella 5.0	30.8% (8/26)	25.0% (3/12)	35.7% (5/14)	.56
Impella CP	65.4% (17/26)	66.7% (8/12)	64.3% (9/14)	.90
Impella LD	3.8% (1/26)	8.3% (1/12)	0% (0/14)	.27
ICU stay, d	9.7 ± 10.72 (245)	10.5 ± 10.70 (136)	8.7 ± 10.73 (108)	.17
Duration of index hospitalization, d	12.2 ± 12.86 (298)	14.2 ± 12.28 (152)	10.1 ± 13.17 (145)	.005

Categorical data are expressed as percentage (numerator/denominator). Continuous data are expressed as mean ± SD (denominator).

ECMO, extracorporeal membrane oxygenation; IABP, intraaortic balloon pump; ICU, intensive care unit; MCS, mechanical circulatory support; PA, pulmonary artery; PCI, percutaneous coronary intervention; VAD, ventricular assist device.

a Values derived from chart review.

b Additional MCS or inotropes/vasopressors post-Impella placement and listed above are per the first 24 hours post-Impella placement. Further therapies delivered after 24 hours were beyond the scope of the data collected for the 24-hour assessment and SCAI shock classification.

Treated vessel and lesion characteristics are presented in Supplemental Table S1. Responders had more lesions treated (2.1 vs 1.7; *P* = .0086), were less likely to have a single vessel treated (55.0% vs 66.9%; *P* = .044), and were more likely to have the left anterior descending artery treated (75.4% vs 65.2%; *P* = .045). Baseline Thrombosis in Myocardial Infarction flow was less likely to be 0 for responders (48.1% vs 62.8%; *P* = .037).

### Kaplan-Meier analyses and clinical outcome

**Figure 1:**
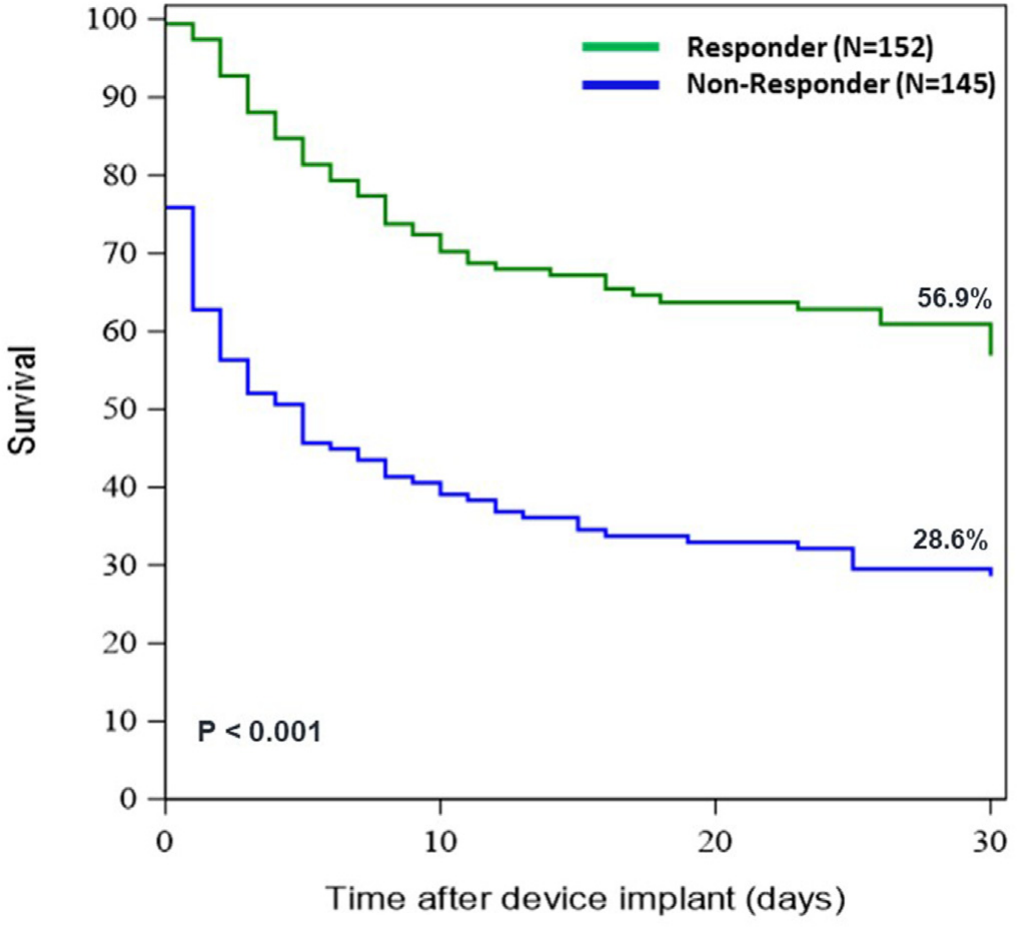
Kaplan-Meier survival curve estimates in Society of Cardiovascular Angiography & Interventions shock stage E patients who improved in shock stage within 24 hours of initiation of Impella support (responders) and those who remained in stage E (nonresponders).

Kaplan-Meier survival curve analysis estimated 30-day survival at 56.9% and 28.6% in the responder and nonresponder cohorts, respectively (*P* < .001; Figure 1). Major adverse cerebrovascular and cardiac event rates through discharge, 30 days, and 90 days were significantly lower in responders (*P* < .0001 for all), driven primarily by lower mortality (Table 5). Responders had a significantly higher rate of cerebrovascular accident/stroke/transient ischemic attack at discharge, 30 days, and 90 days (*P* = .04, .02, and .02, respectively).

**Table 5:** MACCE through 90 days.

	SCAI stage E (N = 298)	Responder (n = 152)	Nonresponder (n = 145)	*P* value
MACCE (in-hospital)	58.1% (173/298)	45.4% (69/152)	71.0% (103/145)	<.001
Death	53.4% (159/298)	38.2% (58/152)	69.0% (100/145)	<.001
MI	1.0% (3/298)	1.3% (2/152)	0.7% (1/145)	.59
CVA/stroke/TIA	6.4% (19/298)	9.2% (14/152)	3.5% (5/145)	.04
Coronary revascularization/emergent CABG	1.7% (5/298)	2.0% (3/152)	1.4% (2/145)	.69
MACCE (30 days)	65.3% (171/262)	54.0% (68/126)	75.6% (102/135)	<.001
Death	59.5% (156/262)	44.4% (56/126)	73.3% (99/135)	<.001
MI	1.2% (3/262)	1.6% (2/126)	0.7% (1/135)	.52
CVA/stroke/TIA	7.3% (19/262)	11.1% (14/126)	3.7% (5/135)	.02
Coronary revascularization/emergent CABG	1.9% (5/262)	2.4% (3/126)	1.5% (2/135)	.60
MACCE (90 days)	70.2% (174/248)	58.3% (70/120)	81.1% (103/127)	<.001
Death	64.5% (160/248)	49.2% (59/120)	78.7% (100/127)	<.001
MI	1.6% (4/248)	1.7% (2/120)	1.6% (2/127)	.95
CVA/stroke/TIA	7.7% (19/248)	11.7% (14/120)	3.9% (5/127)	.02
Coronary revascularization/emergent CABG	2.4% (6/248)	3.3% (4/120)	1.6% (2/127)	.37

Values are percentage (numerator/denominator).

CABG, coronary artery bypass grafting; CVA, cerebrovascular accident; MACCE, major adverse cardiac and cerebrovascular events; MI, myocardial infarction; TIA, transient ischemic attack.

### Predictors of responsiveness

Univariate and multivariable logistic regression analyses were conducted to identify patient and procedural factors predictive of responsiveness (defined as improvement from pre-Impella SCAI shock stage to ≤ 24-hour SCAI shock stage). Variables assessed in univariate analysis are presented in Supplemental Table S2. Those variables identified as potential predictors in univariate analysis were included in the multivariable model, except those variables with significant data missingness, which included pre-PCI Thrombosis in Myocardial Infarction Flow 0, baseline lactate, and baseline pH (Supplemental Table S2), with baseline pH showing a particularly strong, and statistically significant, association with responsiveness. Duration of shock from onset to Impella support approached significance in univariate analysis (*P* = .06) but was not included in the multivariable model, owing to concerns that this data point was not reliably estimated in cVAD, owing to a large proportion of patients transferred from other institutions.

In multivariable analysis using a forward fit model, 6 variables remained in the final model, with 4 identified as statistically significant predictors of responsiveness (Table 6). The single most predictive variable of Impella responsiveness was the number of vasoactive agents used during Impella support, with each additional agent associated with a 0.33 OR for responsiveness (95% CI, 0.23-0.46; *P* < .0001). It is important to note that increased use of vasoactive agents may be an attempt to combat a perceived unresponsiveness to initial therapy. Number of lesions treated during revascularization procedure was also predictive of responsiveness, with each additional lesion treated associated with a 1.5 OR (95% CI, 1.05-2.14; *P* = .0095), as was Impella placement pre-PCI (1.92 OR; 95% CI, 0.90-4.13; *P* = .03). Whereas, WBC was predictive of a lower likelihood of responsiveness (0.93 OR; 95% CI, 0.87-0.99; *P* = .048).

**Table 6:** Multivariable regression analysis, predictors of responsiveness.

Variable	Odds ratio (95% CI)	*P* value
Impella pre-percutaneous coronary intervention	1.92 (0.90-4.13)	.03
Any cardiopulmonary resuscitation	0.36 (0.12-1.08)	.11
No. of inotropes/vasopressors prior to Impella support	0.76 (0.55-1.06)	.10
White blood cell count	0.93 (0.87-0.99)	.048
No. of inotropes/vasopressors during Impella support	0.33 (0.23-0.46)	<.001
No. of lesions treated	1.50 (1.05-2.14)	.009

A total of 199 patients contributed data for the multivariable model. A forward fit approach was used, with all variables associated with a *P* value < .15 in univariate regression analyses for the outcome of interest (improvement in SCAI shock stage within 24 hours) included in the multivariable model. Of 11 independent variables entered into the model, 6 remained in the final model.

Four data points with *P* < .15 in univariate regression analyses (pH, lactate, TIMI flow 0 pre-PCI, and duration of shock from onset to Impella support) were not included in the multivariable model due to limited sample size with data for these variables, which have restricted the sample size for the multivariable model. The univariate analysis findings for these variables are presented in Supplemental Table S2. Duration of shock restricted sample size and additionally, there were concerns that this variable could not be reliably estimated in a real-world registry with 41% transfer patients and an imprecise data point. The output for all variables run in univariate analyses can be viewed in Supplemental Table S2.

CPR, cardiopulmonary resuscitation; PCI, percutaneous coronary intervention.

Kaplan-Meier survival curve analyses were conducted to assess whether survival benefit was observed with the predictors of pre-PCI Impella (compared to intra-Impella/post-PCI Impella) and multiple lesions treated (Supplemental Figure S3). Pre-PCI Impella was associated with significantly higher survival through 30 days in SCAI E patients (50.6% vs 36.3%; *P* = .03), though survival was similar in those with multiple lesions vs single lesion treated (45.9% vs 43.9%; *P* = .60).

## Discussion

In this analysis of AMICS patients undergoing revascularization with Impella support in the multicenter, observational RECOVER III study, we found that stage E patients who improved in SCAI shock staging within 24 hours of Impella support were similar in baseline and admission characteristics to stage E patients who did not improve in shock staging, but differed significantly in treatment strategy. Responders were more likely to have multiple lesions treated, receive Impella pre-PCI, and have fewer vasoactive agents used during Impella support. Responders had significantly higher early survival.

In the last 5 years, the development of the SCAI shock classification system has been widely endorsed by several societies including the American College of Cardiology and American Heart Association,^8^ and is increasingly utilized by clinicians to predict clinical outcomes and guide the determination of appropriate therapeutic strategy (or necessity for patient transfer). The value of serial SCAI stage assessments within the first 24 hours has been demonstrated in recent years in multiple studies.^9^^,^^10^ In the first prospective validation of the SCAI shock classification schema, Baran et al^10^ found that the SCAI shock stage at 24 hours adds significant prognostic value to the baseline assessment. Our initial RECOVER III analysis with SCAI shock staging showed similar results, with a multivariable model of SCAI shock stage classifications at pre-Impella and ≤24 hours of Impella support determining that only SCAI stage E at 24 hours was a significant predictor for early mortality.^6^ Early and more frequent SCAI shock stage classification may provide even more discriminatory value for early clinical outcomes. A recent analysis by Jentzer et al^9^ found that serial SCAI shock classification every 4 hours post cardiac intensive care unit admission was highly predictive of in-hospital mortality, and notably, although admission, minimum, and maximum SCAI shock stages all were predictive of in-hospital mortality, the average SCAI shock stage across that 24-hour window had the most predictive value. Similarly, noting the low availability of lactate values in both this study and other real-world registries and database analyses, we recommend more routine measurement of lactate upon entry to the cath laboratory, with remeasurement roughly every 4 hours until normal. Furthermore, we recommend measurement of invasive hemodynamics prior to exit from the cath laboratory, as forwarded by Basir et al^4^ and the NCSI, in order to identify whether there is a need for escalation of hemodynamic support to an Impella 5.5, or significant right ventricular dysfunction necessitating right-sided Impella support.

Of the 298 RECOVER III patients classified as those in SCAI stage E at pre-Impella assessment, roughly half improved within 24 hours of Impella initiation and half remained in stage E, with a dramatic difference in early survival between those who improved vs those who did not. This analysis sought to identify what differentiated responders from nonresponders. We found that these 2 cohorts had similar presentations, with no significant differences in baseline patient characteristics and medical history. The level of hemodynamic embarrassment was similar in the 2 cohorts, with no significant differences in blood pressure. Invasive hemodynamic data from a pulmonary artery catheter were not readily available. Hence, a comprehensive comparison of hemodynamic compromise in each group was not possible. This may overestimate procedural and postprocedural factors as predictors of responsiveness over baseline factors. Furthermore, lack of available data on the duration of cardiac arrest and status postarrest ultimately led physicians assigning shock stages to classify both in-hospital and out-of-hospital cardiac arrest as criteria for SCAI shock stage E, though in-hospital arrest was accompanied by at least 1 other criterion for stage E. Nonresponders had a significantly lower pH at baseline and elevated WBC, which may indicate some early systemic inflammatory response or superimposed infection, or higher baseline stress. There was also a numerically higher baseline lactate in nonresponders, though there was significant data missingness for this variable. Admission characteristics were similar, with the exception that responders appeared to have a longer duration of shock prior to Impella initiation.

Significant differences between these 2 cohorts emerged in treatment strategy. Responders were more likely to have a pulmonary artery catheter placed, and to receive Impella pre-PCI. Responders received fewer vasoactive agents prior to Impella initiation, and also received fewer agents during Impella support—though this may be reflective in itself of the patient’s responsiveness to therapy. Responders also had a significantly higher number of lesions treated. These treatment characteristics (notably, pre-PCI Impella, more lesions treated, and fewer vasoactive agents being administered) emerged as significant predictors for improvement in the SCAI shock stage within 24 hours. Ultimately, those stage E patients who responded had significantly higher survival (56.9% vs 28.6% survival through 30 days).

These findings emphasize the importance of adhering to the best practices highlighted in the NCSI, in which 70% of patients received Impella pre-PCI, 90% had use of pulmonary artery catheter, and a protocolized escalation and weaning algorithm emphasized the need to avoid escalating doses of vasoactive drugs. Utilizing such an approach, the investigators reported a 71% survival to discharge,^4^ a remarkably higher survival rate than historically reported for AMICS, with survival rates often reported between 50% to 60%.^11^, ^12^, ^13^

In an analysis of the early cVAD registry conducted from 2009 to 2014, investigators assessed the association of pre-PCI Impella with survival to discharge in AMICS patients. They found a significantly higher survival if Impella was used pre-PCI (*P* = .04).^14^ These observations ultimately led to the best practices protocol implemented in the NCSI.

In an NCSI analysis evaluating the impact of increasing vasoactive agents on survival, investigators found a significant decrease in survival with each additional vasoactive agent that was administered. Increasing vasoactive agents were independently associated with mortality in regression analysis, both unadjusted and after adjustment for baseline patient factors, with 1 agent being associated with a 0.22 OR for survival (compared to a reference of 0 vasopressors) and 2+ agents being associated with a 0.18 OR (*P* = .02 and .029, respectively).^15^ Interestingly, in an interim analysis of the RECOVER III registry, Shah et al^16^ reported that increasing use of vasoactive agents was the strongest predictor of in-hospital mortality in women (3.03 OR, *P* = .01), though not similarly seen in men (OR 1.18, *P* = .25). Thus, our analysis provides further support for clinicians to continue to utilize these best practices. Even in patients who present with stage E shock, our analysis demonstrates that pre-PCI Impella and the use of fewer vasoactive agents during Impella support were both significant predictors of responsiveness and survival through 30 days. These data also support the finding that continued need for high doses or multiple vasoactive agents while on support, or the need to escalate support, identify a patient population that is nonresponding and associated with higher mortality.

Importantly, our analysis demonstrates that good clinical outcomes are achievable with Impella support and revascularization even in stage E patients, patients who are often considered unsalvageable, or thought to be more appropriate for treatment with extra-corporeal membrane oxygenation.^17^ The DanGer Shock randomized controlled trial (RCT) demonstrated definitively that Impella significantly improves survival in patients with AMICS, whereas prior RCT such as the intra-aortic balloon pump-SHOCK II and ECLS-SHOCK demonstrated no similar benefit with intra-aortic balloon pump or extra-corporeal membrane oxygenation.^18^, ^19^, ^20^ Notably, these outcomes seen in the DanGer Shock trial were achieved in a study population treated between 2013 and 2023, with much of the study enrollment period occurring before the identification of the best practices we have outlined above. Although DanGer Shock constitutes the first RCT demonstrating a proven treatment strategy for AMICS, it is now incumbent upon clinicians to continue to assess and refine the best practices that will improve outcomes further in this critically ill population, as well as understudied patient subgroups.

### Limitations

SCAI shock classification in RECOVER III was not performed in real-time but retrospectively, and this analysis was conducted exclusively on SCAI stage E patients, which the SCAI shock stage expert consensus writing group acknowledged is the most difficult group to assess as such, retrospectively. Furthermore, this analysis was conducted on a real-world data set, with some data missingness for variables to conduct SCAI shock stage classification, such as lactate and pH, and therefore we could not comprehensively compare the level of baseline hemodynamic compromise in each group. We are further limited by a lack of data granularity on the duration of cardiac arrest, and postarrest patient status, necessitating that in-hospital and out-of-hospital arrest be considered as criteria (in combination with at least 1 other factor) for SCAI shock stage E. In the future, the use of machine learning algorithms may be able to meaningfully assist and improve bedside shock classification, though near-term barriers still exist, as outlined in a recent comprehensive review by Zweck et al.^21^

## Conclusion

Approximately 50% of AMICS patients who presented in stage E shock improved to stage D or E within 24 hours. These responders had significantly improved 30-day survival. Predictors of early responsiveness were mostly related to shock treatment strategy and not underlying baseline characteristics. Our results provide further evidence that the use of serial SCAI shock stages, at initial assessment, provides more meaningful prognostic information.
