# Supplementary material for: Acute Myocardial Infarction and Stage E Shock: Insights From the RECOVER III Study
Source: J Soc Cardiovasc Angiogr Interv. 2025 Jan 7;4(2):102462. doi: 10.1016/j.jscai.2024.102462 (PMC11916721; doi:10.1016/j.jscai.2024.102462)
Supplement: Supplementary Figures and Table [file mmc1.docx]

**
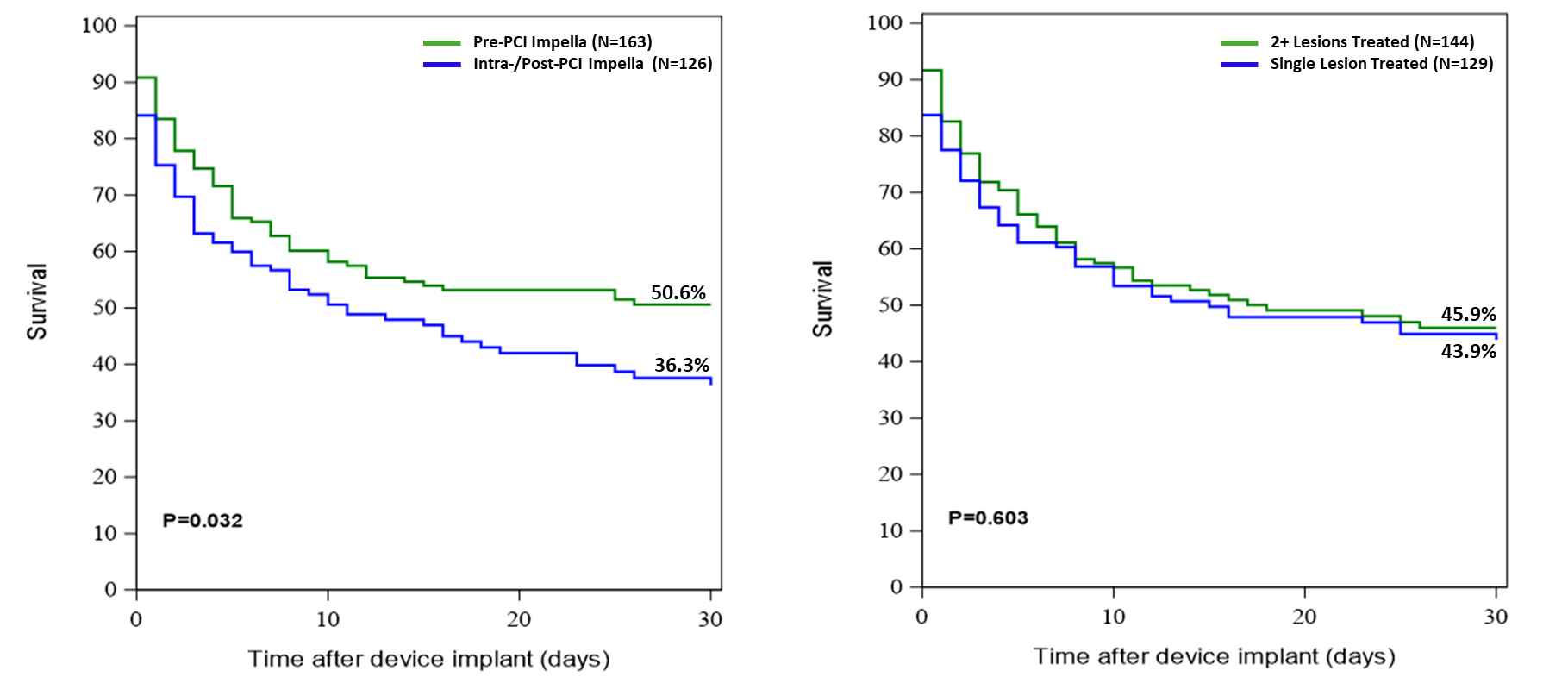
**

**Supplemental Figure S1.** Kaplan Meier curve estimate through 30 days in A) SCAI Stage E patients who received Pre-PCI Impella (N=163) compared to those who received intra-/post-PCI Impella (N=126); B) SCAI Stage E patients who had multiple lesions treated (N=144) compared to those with a single lesion treated (N=129).

| **Supplemental Table S1.** Treated vessel and lesion characteristics | | | | |
| --- | --- | --- | --- | --- |
|  | **SCAI stage E (N=298)** | **Responder (N=152)** | **Non-Responder (N=145)** | **P-value** |
| Number of vessels treated |  |  |  |  |
| 1 | 60.9 % (167/274) | 55.0 % (77/140) | 66.9 % (89/133) | 0.04 |
| 2 | 28.5 % (78/274) | 32.9 % (46/140) | 24.1 % (32/133) | 0.11 |
| 3 | 10.6 % (29/274) | 12.1 % (17/140) | 9.0 % (12/133) | 0.40 |
| Target vessel (patient-based) |  |  |  |  |
| GRAFT | 3.6% (10/275) | 4.2% (6/142) | 3.0% (4/132) | 0.75 |
| LAD | 70.2% (193/275) | 75.4% (107/142) | 65.2% (86/132) | 0.045 |
| LCX | 36.7% (101/275) | 38.7% (55/142) | 34.8% (46/132) | 0.42 |
| LM | 19.6% (54/275) | 22.5% (32/142) | 16.7% (22/132) | 0.19 |
| RCA | 35.6% (98/275) | 35.9% (51/142) | 34.8% (46/132) | 0.74 |
| No. lesions treated | 1.9± 1.22 (273) | 2.1± 1.42 (139) | 1.7± 0.94 (133) | 0.009 |
| Target lesion (patient-based, can have multiple locations) |  |  |  |  |
| Distal | 27.4% (74/270) | 27.7% (39/141) | 27.3% (35/128) | 0.76 |
| Mid | 53.7% (145/270) | 62.4% (88/141) | 44.5% (57/128) | 0.001 |
| Ostial | 8.9% (24/270) | 9.9% (14/141) | 7.8% (10/128) | 0.46 |
| Prox | 71.9% (194/270) | 70.2% (99/141) | 73.4% (94/128) | 0.96 |
| TIMI flow pre PCI (minimum) |  |  |  |  |
| 0 | 54.7 % (111/203) | 48.1 % (52/108) | 62.8 % (59/94) | 0.04 |
| 1 | 9.4 % (19/203) | 12.0 % (13/108) | 6.4 % (6/94) | 0.17 |
| 2 | 17.7 % (36/203) | 18.5 % (20/108) | 17.0 % (16/94) | 0.78 |
| 3 | 18.2 % (37/203) | 21.3 % (23/108) | 13.8 % (13/94) | 0.17 |
| TIMI flow post PCI (minimum) |  |  |  |  |
| 0 | 3.6 % (8/224) | 4.1 % (5/121) | 2.9 % (3/102) | 0.63 |
| 1 | 1.3 % (3/224) | 0.8 % (1/121) | 2.0 % (2/102) | 0.46 |
| 2 | 8.0 % (18/224) | 6.6 % (8/121) | 9.8 % (10/102) | 0.38 |
| 3 | 87.1 % (195/224) | 88.4 % (107/121) | 85.3 % (87/102) | 0.49 |
| Categorical data is expressed as percentage (numerator/denominator). | | | | |

| **Supplemental Table S2.** Univariate regression analyses, predictors of responsiveness | | |
| --- | --- | --- |
| **Variable** | **OR (95% CI)** | **P value** |
| Age | 1.00 (0.98-1.02) | 0.72 |
| LVEF | 0.99 (0.97-1.02) | 0.53 |
| BSA | 0.96 (0.87-1.06) | 0.41 |
| Diabetes | 0.91 (0.57-1.47) | 0.71 |
| Prior CABG | 1.61 (0.65-3.96) | 0.30 |
| Prior PCI | 1.07 (0.63-1.82) | 0.80 |
| Renal insufficiency | 1.35 (0.70-2.59) | 0.38 |
| pH ≤ 7.1 | 0.36 (0.19-0.70) | 0.002 |
| Lactate | 0.94 (0.88-1.00) | 0.06 |
| Creatinine | 0.99 (0.97-1.01) | 0.22 |
| MAP | 1.00 (0.99-1.01) | 0.80 |
| SBP | 1.00 (0.99-1.01) | 0.96 |
| Hospital transfer | 0.82 (0.49-1.36) | 0.43 |
| Cardiogenic shock on admission | 1.03 (0.60-1.77) | 0.92 |
| Duration of shock from onset to Impella (ordinal)* | 1.37 (0.99-1.90) | 0.06 |
| Hypoxic brain injury | 0.57 (0.23-1.44) | 0.23 |
| End organ hypoperfusion | 1.46 (0.73-2.94) | 0.29 |
| OHCA | 0.89 (0.56-1.44) | 0.65 |
| IHCA | 0.85 (0.54-1.34) | 0.48 |
| CPR at time of Impella | 0.64 (0.35-1.16) | 0.14 |
| STEMI | 0.73 (0.40-1.33) | 0.31 |
| No. inotropes/vasopressors prior to Impella | 0.70 (0.58-0.85) | <0.001 |
| MCS prior to Impella | 0.95 (0.55-1.64) | 0.85 |
| Duration of Impella support | 1.00 (1.00-1.01) | 0.43 |
| Impella pre-PCI | 1.84 (1.15-2.95) | 0.01 |
| Door to support < 90 min | 1.07 (0.62-1.82) | 0.82 |
| Impella 2.5 | 2.00 (0.78-5.11) | 0.15 |
| Emergent PCI | 1.25 (0.74-2.10) | 0.41 |
| LM disease | 1.33 (0.77-2.30) | 0.31 |
| PAC use | 2.29 (1.15-4.58) | 0.02 |
| TIMI 0 pre-PCI | 0.55 (0.31-0.97) | 0.04 |
| TIMI <3 post-PCI | 0.76 (0.35-1.66) | 0.49 |
| WBC | 0.94 (0.91-0.98) | 0.007 |
| Heart Rate | 0.99 (0.98-1.00) | 0.05 |
| No. inotropes/vasopressors during Impella | 0.22 (0.12-0.39) | <0.001 |
| No. vessels treated | 1.39 (0.97-1.99) | 0.07 |
| No. lesions treated | 1.33 (1.07-1.67) | 0.01 |
| LAD target vessel | 1.64 (0.97-2.76) | 0.07 |
| BSA, body surface area; CABG, coronary artery bypass grafting; CPR, cardiopulmonary resuscitation; IHCA in-hospital cardiac arrest; LAD, left anterior descending; LVEF, left ventricular ejection fraction; MAP, mean arterial pressure; OHCA, out of hospital cardiac arrest; OR, odds ratio; PCI, percutaneous coronary intervention; SBP, systolic blood pressure; STEMI, ST-elevation myocardial infarction; WBC, white blood cell. | | |


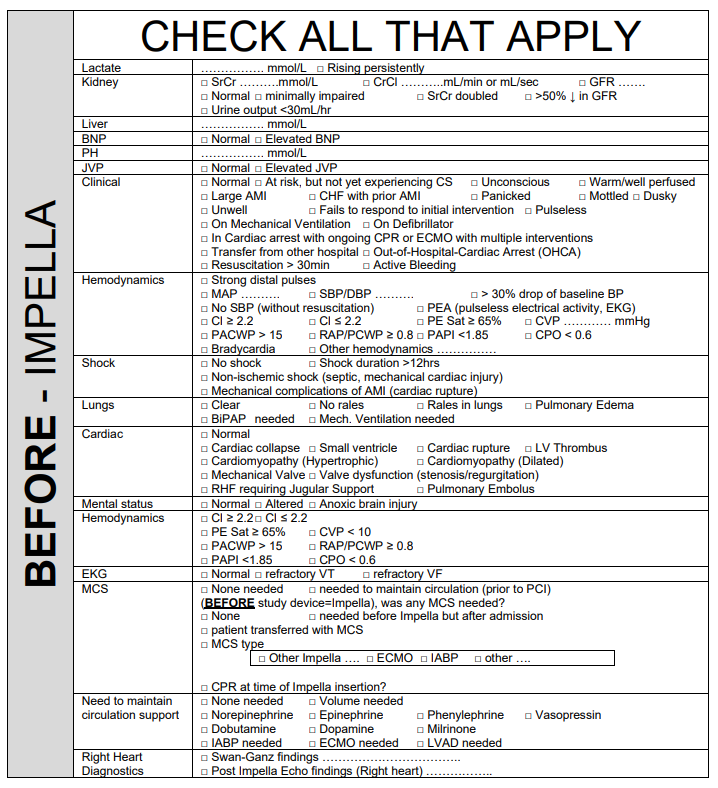


**Supplemental Figure S2**. Data collection tool used for baseline.


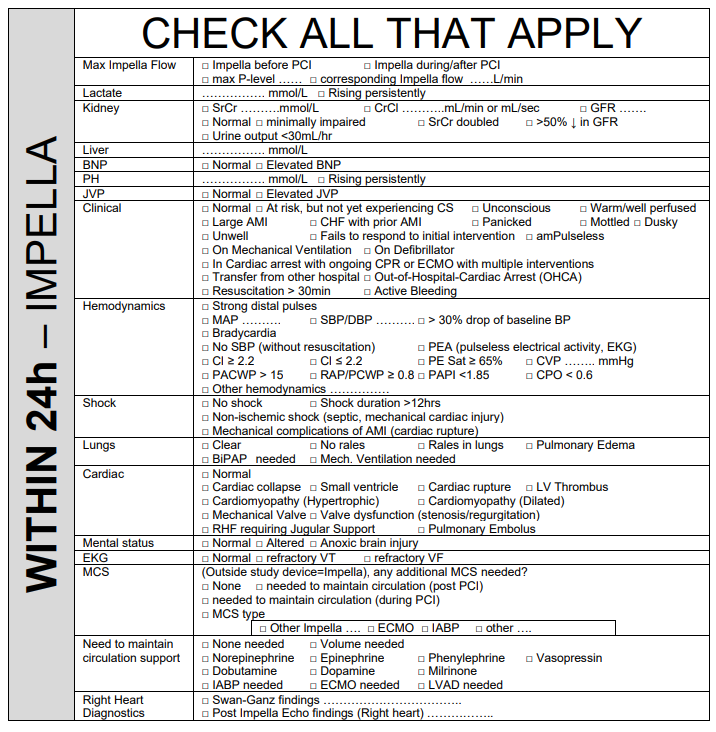


**Supplemental Figure S3**. Data collection tool used for repeat assessment within 24 hours.
